# Peer-to-peer lending and bias in crowd decision-making

**DOI:** 10.1371/journal.pone.0193007

**Published:** 2018-03-28

**Authors:** Pramesh Singh, Jayaram Uparna, Panagiotis Karampourniotis, Emoke-Agnes Horvat, Boleslaw Szymanski, Gyorgy Korniss, Jonathan Z. Bakdash, Brian Uzzi

**Affiliations:** 1 Northwestern Institute on Complex Systems, Northwestern University, Evanston, Illinois, United States of America; 2 Indian Institute of Management, Bangalore, Karnataka, India; 3 Dept. of Physics, Applied Physics and Astronomy, Rensselaer Polytechnic Institute, Troy, New York, United States of America; 4 Social and Cognitive Networks Academic Research Center, Rensselaer Polytechnic Institute, Troy, New York, United States of America; 5 Department of Communication Studies, School of Communication, Northwestern University, Evanston, Illinois, United States of America; 6 Dept. of Computer Science, Rensselaer Polytechnic Institute, Troy, New York, United States of America; 7 US Army Research Laboratory, Aberdeen Proving Ground, Maryland, United States of America; 8 US Army Research Laboratory South Field Element at the University of Texas Dallas, Dallas, Texas, United States of America; 9 Texas A&M University-Commerce, Commerce, Texas, United States of America; Rutgers The State University of New Jersey, UNITED STATES

## Abstract

Peer-to-peer lending is hypothesized to help equalize economic opportunities for the world’s poor. We empirically investigate the “flat-world” hypothesis, the idea that globalization eventually leads to economic equality, using crowdfinancing data for over 660,000 loans in 220 nations and territories made between 2005 and 2013. Contrary to the flat-world hypothesis, we find that peer-to-peer lending networks are moving away from flatness. Furthermore, decreasing flatness is strongly associated with multiple variables: relatively stable patterns in the difference in the per capita GDP between borrowing and lending nations, ongoing migration flows from borrowing to lending nations worldwide, and the existence of a tie as a historic colonial. Our regression analysis also indicates a spatial preference in lending for geographically proximal borrowers. To estimate the robustness for these patterns for future changes, we construct a network of borrower and lending nations based on the observed data. Then, to perturb the network, we stochastically simulate policy and event shocks (e.g., erecting walls) or regulatory shocks (e.g., Brexit). The simulations project a drift towards rather than away from flatness. However, levels of flatness persist only for randomly distributed shocks. By contrast, loss of the top borrowing nations produces more flatness, not less, indicating how the welfare of the overall system is tied to a few distinctive and critical country–pair relationships.

## Introduction

The “flat-world hypothesis” is an idea of a new “level” playing field where global economic equality gradually improves, is seductive [1]. Models of financial markets suggest that international capital flows are reaching more countries [2] and dominating national institutional policies [3], thereby laying a groundwork for global equality in access to capital that can promote new possibilities for prosperity among the world’s poor [4–7]. However, others have countered that outside of a handful of cities/countries the vast majority of economic activities (e.g., institution and government investment, web traffic, and telecommunications) have remained domestic over time [8, 9]. As crowdfinancing grows is it a flat-world mechanism for creating opportunities for the world’s poor, or does it follow the biased-patterns exhibited by other established economic activities and mechanisms? The Lucas Paradox [10] indicates that, counterintuitively, the liberalization of international capital regimes has not produced an open club, but rather a rich club–that is, a group of countries with similarly well-developed monetary institutions, cultures, and wealth that display in-group preferences [11] in lending and borrowing, thus restricting capital to poor nations [12–13]. Whether the Lucas Paradox occurs with philanthropic crowdfinancing is an open question and a means for testing the flat-world hypothesis.

New data on global crowdfinancing allows questions to be asked about the role of peer-to-peer lending networks in leveling global capital financial flows and development. Crowdfinancing is a recent innovation. It enables private lenders and borrowers to find and directly interact with one another through a website. Private individuals on the website, from theoretically anywhere around the world, can lend or borrow capital directly from each other. Borrowers put forth their reasons (see S1 File for examples) and make requests for capital directly to lenders; in turn, lenders make their lending decisions free of institutional constraints. In this way, peer-to-peer lending sidesteps the long-standing institutional arrangements and cultural norms that have up to this point characterized lending [14, 15] (see S1 File for a comparison between Kiva and government aid between countries). Crowdfinance offers an alternative and/or supplemental mechanism to more institutionalized forms of foreign aid. The flow of such aid is associated with increased stability, such as reductions in terrorism [16]. However, the success of foreign aid is marred by corruption, political changes, and other factors (e.g., see [17]). The average loan size in crowdfinance is positively associated with lower corruption levels in the country [18]. Thus, crowdfinance provides a potential mechanism for unmediated, direct aid especially if it tends towards “flatness”–that is, one with fewer institutional and cultural biases in lending in terms of opportunities for the poor, over time.

Despite the possibility for crowdfinancing to level the playing field in capital flows, its potential is debated [19] and empirical patterns are largely unknown [20]. One critical association between peer-to-peer lending and global financial flows concerns the flat-world hypothesis [1]. The flat-world hypothesis holds that crowdfinancing counter-balances lending biases, i.e., patterns of preferential lending (higher or lower than expected by chance) activity at the country-pair level, by acting as a functional substitute for capital from traditional lenders and lending institutions ([1], see pp. 492–493). However, the increased interconnectedness may also potentially make the world less flat by reinforcing the existing global or individual level biases [21]. If the flat-world hypothesis is correct, peer-to-peer lending systems should display no preferential attachment of capital flows between lender-borrower pairs [22].

To examine the flat-world hypothesis, we analyzed the total aggregate lending of over half a billion dollars in over 600,000 peer-to-peer loans made on one of the largest and well-regarded crowdfinancing websites in the world, “Kiva” from its inception in 2005 to 2013 [23]. Loans, mostly from private, individual lenders in more than 220 nations and territories were made to private borrowers in 80 countries. The list also includes a few geographical regions and territories. For simplicity, hereafter we refer to them as countries in this paper. Kiva is philanthropic in nature and lenders receive their capital back without interest and borrowers receive loans without paying interest. By comparison, the aggregated (2005–2013) government aid for the same time period involves 48 donor countries making interest-based loans (data from AidData [24], see S1 File). Our study examines three related questions about crowdfinancing. First, to test whether crowdfunding loans are associated with a flatter world, we measure the degree of flatness in the lending system. A flat-world has capital flows that display no preferential attachment between lender–borrower pairs [25]. To quantify “flatness,” we randomly rewire the observed co-country network of loans, which creates a hypothetical Kiva network wherein the propensity for any lender–borrower transactions is no greater than expected by chance. Deviation from the expected null network of flows reflects choice in lending and hence a less flat world [25]. Second, we use regression analysis to predict bias in country–pair transactions based on variables such as GDP, geographical distance that are typically used in gravity models of trade [26, 27]. Although previous studies [20, 28] have investigated the biases associated with lending on Kiva, our study presents a longitudinal analysis for a longer observation window (2005–2013). Since the number of participating borrower countries as well as the transactions have grown significantly in the later years, it becomes important to account for yearly changes in the network as opposed to treating it in a cross-sectional fashion. Nevertheless, some of the factors that we find associated with lending bias are qualitatively consistent with the findings of Burtch et al. [20]. Third, we investigate the potential susceptibility of the Kiva network to shocks that could change the system’s ability potential for flatness. Shocks to lending systems include national policy changes, market collapse, climate change, health or security risks [29], and have been shown to dramatically alter capital flows [30]. We represent these hypothetical changes in the system as the disappearance of network nodes or links [31–33] and then observe their simulated effects on the network structure and its flatness.

## Data

Crowdfinancing networks differ in orientation. Some crowdfinancing systems provide funds in exchange for equity in an investment (e.g., Equitynet.com, CrowdCube.com, Seedrs.com) or supply interest-bearing investments (e.g., Prosper.com). Others, promote interest-free loans, in which no monetary interest is gained by the lender, but contributions are made for the developmental aid of the borrower (e.g., Kiva.com). In addition, other forms of crowdfunding such as funding a project for non-financial returns (Kickstarter) and charity where no return is expected also exist [34]. Our dataset of lenders, borrowers, and loans includes all transactions made on Kiva.com, 2005–2013. Although the average loan size on Kiva is about $700, the vast majority of loan contributions are made in multiples of $25.00 and most contributions are for $25.00 and $50.00. These loans typically support purchases of machinery for petty entrepreneurs, livestock for farmers, or domestic items such as water purification systems that improve living conditions (see S1 File for case examples). For each loan we know the:

1. Time of effectuation
2. Size of the loan
3. Location of the lender (self-reported, coded at the country or territory level)
4. Borrower and the specific Kiva field partner, that is, a representative of Kiva who provides access to computers to potential borrowers, helps them translate or edit their requests for a loan in English, and manages lender-borrower transactions.

**Fig 1:**
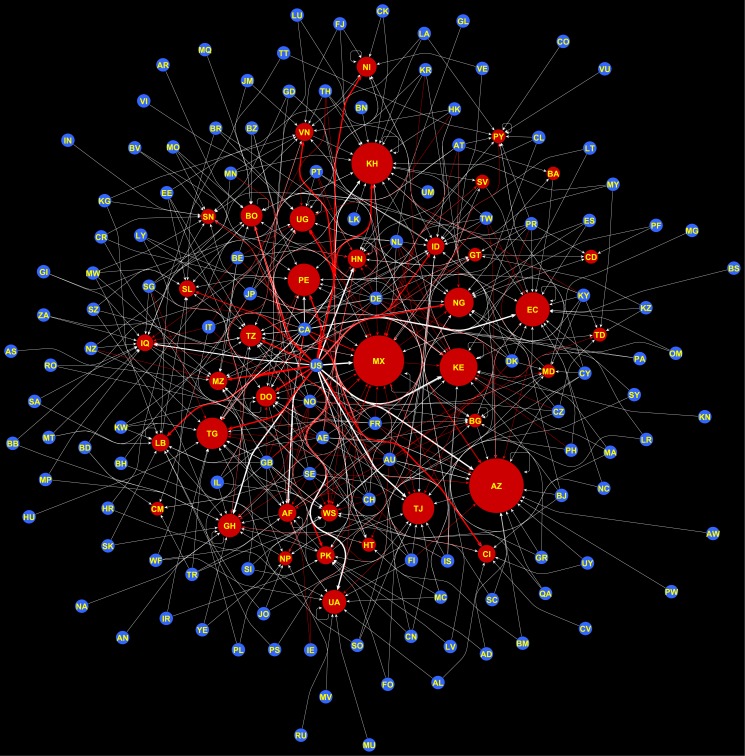
Biased links in the Kiva network. Visualization of positively (colored white) and negatively (colored red) biased links in the Kiva co-country network for 2007. Borrower countries (nodes) are shown in red with size proportional to the total transactions received by that country; whereas, lender countries are shown in blue and all nodes are of the same size. The link thickness corresponds to the actual number of transactions made between the country–pairs (more visualizations from the same year are shown in SI).

**Fig 2:**
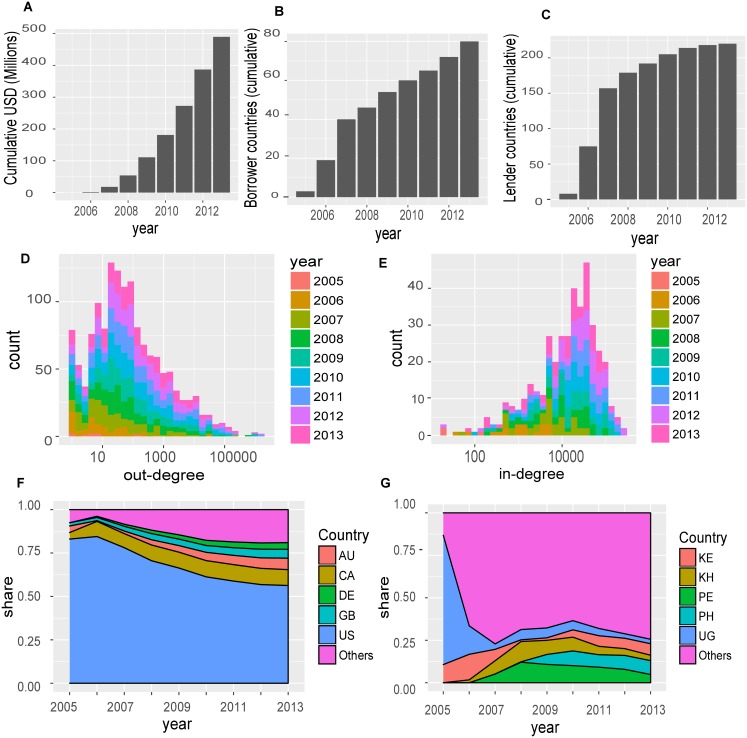
Evolution of the Kiva country network. (A) Total annual money lent through Kiva (cumulative). (B) Cumulative number of borrower and (C) lender countries. Plot (A)–(C) show the rapid growth of Kiva as platform for crowdfinancing both in terms of money lended and level of participation. (D) Histogram of total number of outgoing transactions from countries (out-degree) and (E) histogram of total number of incoming transactions to countries (in-degree), color stacked by year. The X-axis scale is logarithmic, thus, the histograms reflects the skewness of the distributions. (F) Top five lender countries and their share of loans given and (G) top five borrower countries for each year. The US accounts for a major share of the lending activity (> 50%), however the US dominance is decreasing with time as we see increased participation levels from more countries.

We constructed a yearly co-country network aggregated from the country-to-country transactions (an example is shown in Fig 1). Loans to compatriots (i.e., self-loops in the network), are allowed. Fig 2(A)–2(G) summarizes the growth of the co-country network and shows that money lent in the form of loan contributions and the number of participating borrower and lender nations grew dramatically on Kiva between 2005 and 2013. A few lender countries account for a large portion of the loan transactions. Fig 2(F) shows the top 5 lender countries and their share of transaction volumes by year. It can be seen that these 5 countries together account for about 80% of all observed contributions with the US alone being responsible for more than half of the contributions. The top 5 borrower countries benefit from a large portion of the total contribution, but there is no clear outlier and there are many countries with a similar share of received contributions (Fig 2[G]). The same trend can be seen in terms of the degree distribution of the network. The in-degree (out-degree) of a country is the sum of transactions made to (by) that country. Fig 2(D) and 2(E) show that both in-degree and out-degree distributions are skewed (log scale), but the out-degree distribution is highly skewed (i.e., a few lender countries provide a very large portion of the observed transactions).

## Results

To analyze the structural property of the network, we used degree-preserving network randomization, a common technique for assessing the statistical significance of observed network properties, including biased links between nodes [35–38]. Using the randomization method for weighted (multiedge) networks, we generate many synthetic networks by randomly rewiring the loan transactions in the observed network [39] while preserving the total transactions made to and from, for each country (i.e., in- and out-degree of every node). Many synthetic networks provide a distribution of every bilateral exchange, giving an expected mean and standard deviation across all links in the network, which are used to determine how far observed relationships are from expected values (see S1 File). A comparison between the null model and the observed data enables us to identify country-level lending biases in this network–that is, which countries have a lending–borrowing relationship that is greater or smaller than expected by chance, where chance theoretically reflects a system without bias [22]. To measure the flatness of the lending network, we count the number of country–pairs (positive as well as negative) where the observed links are statistically different from what is expected using a z-score for each pair of countries. The z-score *z*_*ij*_ of any link *ij* is given by

$$z_{ij}=\frac{O_{ij}-{Ε}_{ij}}{\sigma_{ij}}$$ (1)

where *O*_*ij*_ is the observed number of transactions from a country *i* to country *j*. *E*_*ij*_ and *σ*_*ij*_ are the expected number of transactions and the associated standard deviation according to the null model. For a country–pair, the z-score provides a normalized and relative measure of how far away the observed number of transactions is from what is expected by chance. A pair is classified as biased if its observed number of transactions is 2 standard deviations above or below the null model (*p*<.05).

The flatness is then given by the fraction of unbiased links:

$$flatness=1-\frac{number\;of\;biased\;links}{total\;number\;of\;links}$$ (2)

**Fig 3:**
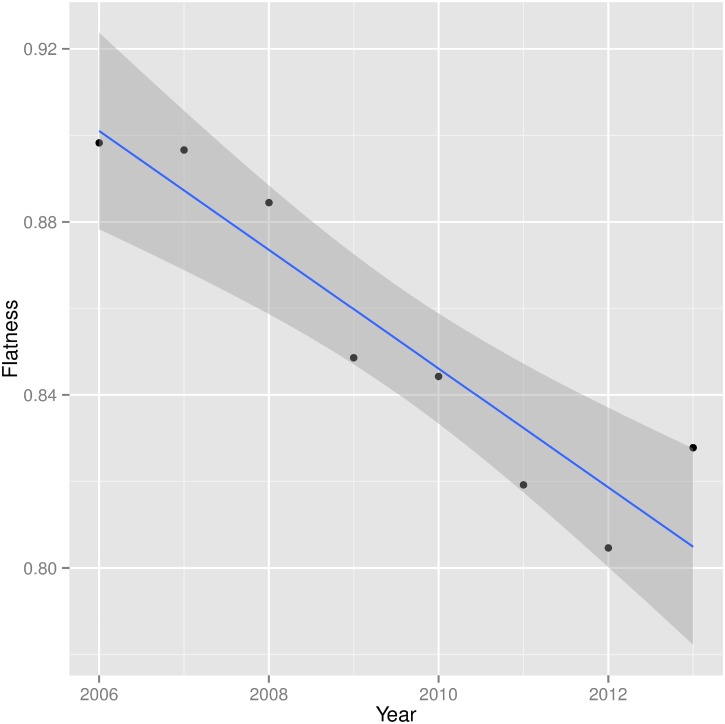
Flatness of the Kiva network. Flatness is defined as the fraction of unbiased country–pairs (|z| ≤ 2) under the null model. The flatness is measured by comparing the observed flow with the expected flow as described in the text. The line fit reveals a trend of decreasing flatness over the considered time frame (2006–2013).

The measured flatness in the year range 2006–2013 is shown in Fig 3 and is systematically decreasing with time. This indicates a statistically significant trend of less rather than more flatness. Between 2006 and 2013 (we drop the year 2005 from this analysis due to the small number of transactions made in that year), the flatness dropped by nearly 10% from its initial value (a detailed comparison of z-score distributions is shown in S1 File).

**Fig 4:**
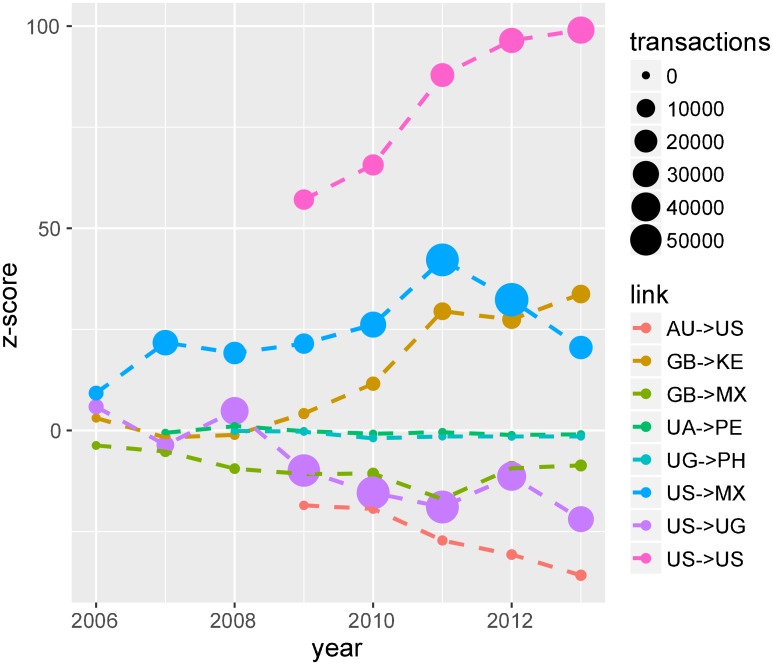
Evolution of link biases. Time series of link level bias measured as z-scores for select links. The size of each dot corresponds to the number of transactions across the corresponding link. Few of these links (e.g., US to MX) are consistently biased while a few are unbiased (contributing to flatness) for all years.

An examination of country–pairs reveals that some pairs show persistent bias (positive as well as negative), whereas others remain unbiased through time. Fig 4 shows the time evolution of z-scores of a few of these country pairs. An example of positive bias (over-lending relative to the null model expectations) in the network is illustrated by loans from the US to Mexico. In the year 2012 there were ~59 k transactions made from the US to Mexico, about 5 k more than expected by the null model (~54 k), which corresponds to a z-score of +32. Loan contributions made to borrowers in US and lenders from other countries usually show a negative bias. For example, transactions from Australia to the US in the same year (2012) were only 639. This observation is much lower than expected, 1,962 transactions with a z-score of –31. However, this is compensated by US-to-US over-lending (self-loop) as shown in Fig 4. Interestingly, within country lending and borrowing (positive bias associated with self-loops) is seen consistently across the whole network and over time.

To further investigate the factors associated with lending bias between nation pairs, we regress the level of lending between nations on factors effecting bilateral international trade with the widely used fixed-effect gravity model [26, 27]. In this model, the level of trade from country *i* to country *j*, *Y*_*ij*_, is modeled as

$$Y_{ij}=G\frac{M_{i}^{\alpha }M_{j}^{\beta }}{d_{ij}^{\gamma }}$$ (3)

where *M*_*i*_ and *M*_*j*_ are the economic masses (e.g., GDP) of *i* and *j*, *d*_*ij*_ is the geographical distance between *i* and *j*, and *G* is a constant. The parameters to be estimated are α, β, and γ, respectively. We aggregate transactions such that each observation *Y*_*ijfy*_ denotes the number of transactions from the lender country *i* to the borrower country *j* involving the Kiva field partner *f* for given year *y*. Field partners are microfinance institutions (e.g., NGOs, schools, or social enterprises) operating in the borrower country and are responsible for connecting borrowers with Kiva, screening them, posting their loan requests online, and disbursing and collecting repayments. Since many country–pairs in our data show zero transactions, the log transformation of the level of bilateral trade typically used in the gravity model is not feasible in our setting. Thus, we ran a second model that appropriately accounts for the skewness in the level of loans between countries by discretizing the dependent variable *Y*_*ijfy*_ into four categories (denoted by *Q*_*ijfy*_) that correspond to “zero”, “low”, “medium”, or “high” levels of lending [40]. We performed a fixed-effects ordered logistic regression on the transformed variable to control for unobserved heterogeneity related to the lender country, borrower country, Kiva field partner, or year. Zero transactions category is the omitted category. The ordered logit and the gravity model produce qualitatively similar results (see S1 File for details).

Per the gravity model, we include four explanatory variables in our regression: (i) the difference of per capita GDP between lender and borrower countries [41]; (ii) the geographical distance between the country–pairs [42]; (iii) the size of the migrant population of borrower country living in the lender country [43]; and (iv) an indicator variable showing that lender country colonized borrower country (1 = yes), which captures common culture and institutional structures [42, 44]. Our model is as follows:

$$Q_{ijfy}=\beta_{1}{GDP\;difference}_{ij}+\beta_{2}{Distance}_{ij}+\beta_{3}{Migration}_{ji}+\beta_{4}{Colony}_{ij}+\varepsilon_{ijfy}$$ (4)

This model (Model 4) unequivocally had the best fit, with an evidence/likelihood ratio of 12.05 × 10^5^ over the next best fit model (Model 3) [45]. The regression findings reported in Table 1 suggest that bilateral transaction volumes in this peer-to-peer lending system reflect general patterns of trade between nations rather than unique peer-to-peer patterns. The per capita GDP difference between countries, migration between county pairs, and the historical presence of a colonial relationship are all positively (odds ratio > 1) and significantly associated with lending volumes, while geographical distance is negatively and significantly associated with the level of lending (odds ratio < 1). These findings suggest that the greater global context within which peer-to-peer lending is embedded impacts crowdfinancing in much the same way that it does other forms of global trade. We also apply the same model on AidData using four categories of country-to-country government aid money (“zero”, “low”, “medium”, “high”) as the outcome variable (see S1 File for more details). The results shown in the last column of Table 1 imply that distance, migration, and colonial tie are associated with level of aid in the same manner. However, much higher odds ratio for migration and colony (compared to Kiva) indicate that these variables have a much stronger association with flow of government aid. Surprisingly for government aid, the effect of per capita GDP difference is not found to be significant (p > 0.05), which in the case of Kiva is shown to be positive and significant (Table 1).

**Table 1:** Fixed-effect ologit estimates of levels of lending between countries. Odds ratio reported for 4 levels of transactions (4 levels of commitment amount in the case of government aid) as defined in the text and S1 File.

	Odds ratio Model 1	Odds ratio Model 2	Odds ratio Model 3	Odds ratio Model 4	Odds ratio Govt. Aid
**GDP (pc) difference**	1.76**	1.73**	1.73**	1.74**	0.99
**Distance**		0.94**	0.94**	0.94**	0.77**
**Migration**			2.47**	2.24**	2.52**
**Colony**				1.41**	12.65**
**AIC**	181781.5	176346.9	162648.1	162624.7	
**BIC**	181950.9	176555.7	162855.0	162772.1	
**Fixed Effects**					
**Year**	Yes	Yes	Yes	Yes	Yes
**Partner**	Yes	Yes	Yes	Yes	-
**Lender (donor) country**	Yes	Yes	Yes	Yes	Yes
**Borrower (recipient) country**	Yes	Yes	Yes	Yes	Yes

**p < 0.05

**Fig 5:**
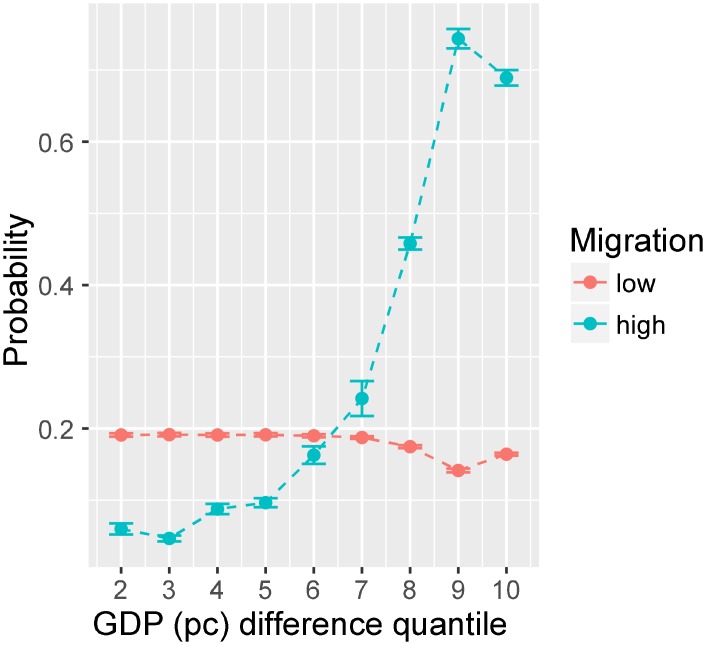
Marginal effects of GDP per capita difference and level of migration. Y-axis measures the probability of observing large numbers of transactions (i.e., the outcome *Q*_*ijfy*_ falling into a “high” category), as a function of GDP difference quantile and for different levels of migration (low vs. high). For low migration the probability shows no increase with GDP difference quantile, but for high migration the probability shows a significant increase–specifically beyond the 50th percentile of GDP difference. The plot shows that migration is only effective when it moves migrants from a low GDP to a high GDP country (which corresponds to direction across a large and positive GDP difference).

To depict these effects in Kiva over the range of the variables, we plot the relationship between transaction flows, GDP difference, and migration from an ordered logistic regression (ologit) using quantiles of GDP difference and high and low migration (split at the median). Fig 5 shows the probability of high transaction volumes (8 to 54,136 transactions) at different quantiles of GDP difference for different levels of migration. The plot shows an increasing trend in lending associated with growing per capita GDP for country–pairs that share a large (above the median) and no significant change for small (below the median) immigrant population. We observe that the effect of GDP difference is weak up to its 60th percentile after which it shows a much stronger impact on loan levels. This suggests that much of the source of bias in the system is keyed to high GDP lenders. Specifically, for lower than 60th percentile, the probability of observing biasedly high-volume transactions is quite small (< 0.2) but grows rapidly for higher percentiles of GDP difference (~ 0.75 at 90th percentile, in the case where migration level is also high). Interestingly, the results show that migration from borrower to lender country only plays a role when the per capita GDP of the lender country is sufficiently higher than that of the borrower country (otherwise migration shows a slight negative association). It can also be seen that higher GDP difference with high migration has a strong positive effect on the transaction volumes, suggesting that the deeply embedded structures that characterize relationships among nations continue to impact the networked systems such as Kiva. These findings indicate that while crowdfinancing may have reduced some biases [46] in the lending system, the greater global context within which peer-to-peer lending is embedded impacts crowdfinancing in much the same way it does other forms of global trade. Factors associated with the magnitude of bias continue to be correlated with lending pair relationships that deviate from flatness.

### Network robustness

World events have the potential to significantly change the Kiva network and lending systems like it. For example, events can impact the nodes or links in the network at random with events being precipitated by unpredictable financial collapses, coups, or natural disasters [30]. Events that drop nations out of the system can be strategically determined by new regulations, policies, or relationship failures. For example, the construction of a wall between the US and Mexico, an embargo, or a Brexit event could reduce or shut down flows in country–pairs [31–33].

To take a first step in trying to capture these network events in an abstract way, we explore key what-if scenarios of how the Kiva network responds to events that disrupt capital flows. Our what-if shocks occur at the country level (affecting a node) or the country–pair link level. For country/node level effects we remove nodes and all their links in four scenarios: (i) random removal of borrower nations, (ii) random removal of lender nations, (iii) removal of nations according to their lending volume (out-degree), and (iv) the removal of nations according to the borrowing volume (in-degree). For link removal, we remove links (i) at random, (ii) with minimal z-score, (iii) maximal z-score, and (iv) maximal transaction volume. Node removal is equivalent to a total edge removal when all the edges of a specific node are removed at the same time. For each reshaped network topology, we compare the new network to its corresponding null model distribution.

**Fig 6:**
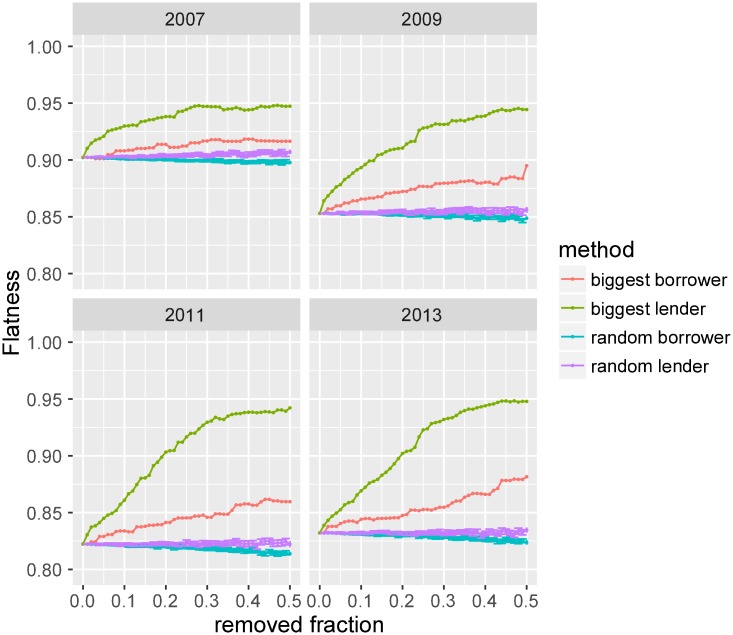
Simulated shocks: The effect of node removal. Change in flatness (defined as the fraction of unbiased links in the network) of the system as a function of removed fraction of nodes for different selection methods and for a few selected years (other years show a similar trend). The error bars correspond to ± 2 standard error for the random borrower and random lender case. The plots suggest that when nodes are removed randomly, the system flatness does not change; however, removing the biggest lenders or borrowers drives the system towards a more flat configuration.

Fig 6 shows the change in flatness as a result of node removal, broken down by year. The x-axis represents the percentage of nodes removed for each of our four scenarios and the y-axis shows the flatness. Our results indicate that the system’s flatness responds differently to random and targeted removal of nodes. The system is remarkably stable when lender or borrower nodes are removed at random. This suggests that shocks that might impact nodes in the network at random are unlikely to change the system properties in regard to flatness. By contrast, the removal of just 10% of nodes targeted by their ranked out- or in-degree rapidly change system dynamics. The removal of only a few big lenders increases flatness quickly in all years. This makes intuitive sense as the big lenders correspond to pairs with larger per capita GDP difference, and therefore, are associated with bias (Fig 5). This increase reaches saturation when the network attains an almost flat configuration. The trend in the elimination of the big borrowers is similar, but not as pronounced. This can potentially be attributed to the difference in out-degree and in-degree distributions. Since the out-degree distribution is more skewed (Fig 2[D]), a few high-degree lender nodes account for a significantly larger portion of observed transactions. Hence, their removal results in the disappearance of more biased links than a high-degree borrower.

Since the in-degrees and out-degrees of nodes are preserved, presence of highly biased connection to a node may force other connections to that node to be biased as well (e.g., under-lending to a country from one or more lender countries balanced by over-lending by others). Due to this interdependency of link biases, a local disruptive change in the network may have cascading effects causing a larger number of links to become biased.

**Fig 7:**
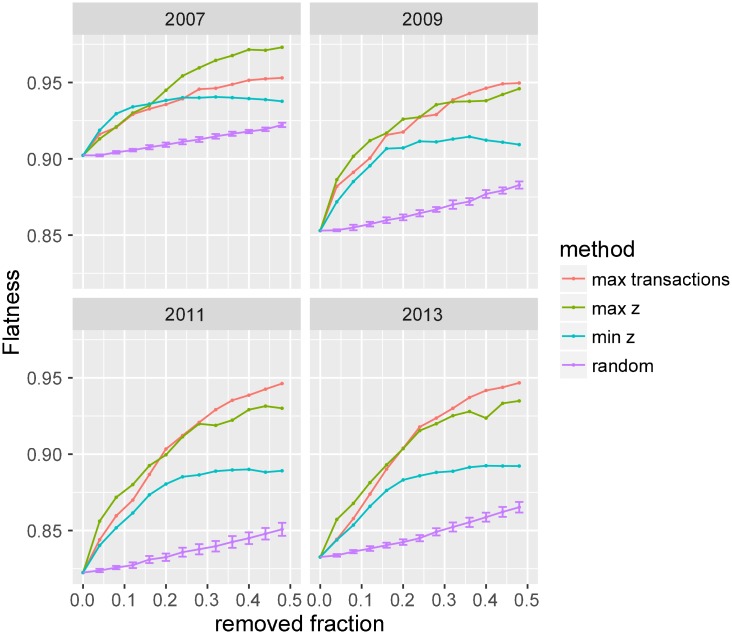
Simulated shocks: The effect of link removal. Change in flatness (defined as the fraction of unbiased links in the network) as a function of removed fraction of links for different selection methods and for a few selected years (other years show a similar trend). The error bars correspond to ± 2 standard error for the random link removal case. Similar to the node removal case, the system flatness does not change appreciably as links are removed randomly. Removing biased links (i.e., maximum or minimum z-scores) and links with maximum transactions makes the system flatter.

The system’s flatness is robust against random removal of edges and increases in flatness with removal of high transaction links (Fig 7). In addition, we investigate the effect of edge removal according to the positivity or negativity of bias. Gradually removing links with strong positive bias causes flatness to increase comparatively to targeting maximum transaction links. This change is more drastic for small fraction of removed links and holds especially for earlier years (when the network was small). Targeting links with strong negative bias results in a weaker increase in flatness. This difference can be understood qualitatively in terms of the slight asymmetry in the z-score distribution. There are more positively biased links than negatively biased links. We also observe that the selection order of link removal based on the highest number of transactions increases most the flatness of the networks for later years (see S1 File).

This sensitivity analysis about system responses to different kinds of removals (nodes or links, random or targeted) reveals that random removal of nodes or links causes little-to-no change in overall flatness of the lending system. However, the flatness increases rapidly as big-players are removed from the network or few important channels of capital flow are blocked. We find that most of the bias in the system is accounted for by these few key countries or country–pairs.

## Discussions and conclusions

Global interconnectedness has raised the possibility that the world is becoming flatter and offering more equality of opportunity worldwide. Online crowdfinancing platforms like Kiva provide alternative channels of capital flow to traditional institutions raising the question as to whether peer-to-peer financing is making the world flatter. To the contrary, we find continued and increasing bias in an inter-country, peer-to-peer crowdfinancing network. This drift towards a less flat world may arise from individual level preferences or global factors. Although crowdfinancing provides a lending platform that connects lenders with borrowers and eliminates conventional intermediaries such as banks, it is the individual lenders who decide whom they give loan to and can often be biased in their decisions. These biases are reinforced and made even stronger by the rapid growth of the crowdfinancing platform itself (“rich gets richer” effect). An example of this growing bias in the crowdfinancing network is seen in the form of self-loops (lenders lending to borrowers in the same country), which are consistently biased in the positive direction. Nonetheless, whether or not these biases will continue to persist in the long run, remains an open question. We explored the effects of hypothetical disruptive events on system-level flatness with simulations and found that the lending network is not vulnerable to random losses of countries or bilateral ties. However, the targeted removal of a few high-volume lenders or high-transaction links could cause the network’s flatness to increase significantly. This implies that the decreasing flatness is not centered on all lending, but on the lending bias of a few giant lenders that skew the overall system. In this way, the flatness of the system is directly linked with the dominance of a few big players. This lending bias by a small number of countries combined with simulation results targeting these lenders, suggest that increasing inequality may be attributed to preferential attachment (“rich get richer”) [47].

Using regression analysis, we identified a few factors associated with preferential lending on this platform. One of the factors that significantly affect lending is economic disparity. Lenders in high-GDP per capita countries show a preference to provide money for low-GDP per capita countries–facilitating capital flow from developed to developing nations. This is important from the point of view of equality as it suggests that Kiva favors links that allow capital to flow from rich to poor countries (a counterexample of Lucas paradox). Other factors effecting lending are migration and colonial past, which are positively associated with lending, along with geographical distance, which has a negative association. Interestingly, these factors also effect other forms of international capital flows in the same manner (the effect size may vary from one system to another), as revealed by analyzing the government aid and shown by previous studies on international trade [26], thus reflecting the embeddedness of crowdfinancing in a larger ecosystem [48, 49]. The association of these factors with trade flow and government aid have to do with reasons that may be logistic (e.g., in trade flows, distance adds to the cost for supplying goods) or sociopolitical (e.g., a colonizing power providing development aid to its past colonies). The same factors that determine the level of bilateral trade or aid are also associated with biasing the capital flows in an online crowdfinancing platform where loan transactions have zero logistic costs. This suggests that while crowdfinancing holds promise to add flatness to the world system of finance, it is embedded in a larger system of stable inequities that limit its effects and influences its development.

## Methods

### Regression specification

The ordered logit is a non-linear model where the dependent variable *Y*_*ijfy*_ (defined as the aggregated number of transactions from the lender country *i* to the borrower country *j* and involving the Kiva field partner *f* for a given year *y*) is converted from a continuous variable to *quantiles* of transaction count between countries (amount of aid between countries in the case of government aid) as the dependent variable with outcomes zero (1), low (2), medium (3), and high (4) based on natural break points in the distribution (see SI). This conversion is done to deal with the non-normality of count data that makes up the dependent variable, the problem caused by log transforming the variables with zero values [50], and also because of the limitation of Poisson models for dealing with this type of data (skewed distribution and containing a large number of zero observations) [51].

We supplement the Kiva data with our explanatory variables: per capita GDP difference (averaged over 2005–2013), inter-country distance, migration, and a categorical variable indicating whether the lender country was a colonizer of the borrower country in the past (a colonial tie). Data on distance between lending and borrowing countries and the presence of absence of colonial past relationships between countries were obtained from the GeoDist data of CEPII, Research and Expertise on the World Economy [42]. Country per capita GDP data were obtained from the World Bank’s World Development Indicators. Finally, data about the number of immigrants between countries came from 2010 estimates of the International Migrant Stocks of the United Nations population division [43]. Since the data are obtained from different sources, after merging, our number of observations is reduced from 174,468 to 140,418 due to availability of data. In addition, the model considers the fixed effects of lender country, borrower country, field partner, and year. (See S1 File for a summary of the dependent and the independent variables and correlations among them.) We check the robustness of our model by comparing it to other models that use a subset of explanatory variables. The model we use corresponds to the optimal set of Akaike information criterion (AIC) and Bayesian information criterion (BIC) statistics [52] (Table 1). To test for multicollinearity among explanatory variables, VIF statistics were checked and found to be satisfactorily low.

### Node removal

Starting from the original observed network, we remove a node (or a set of nodes), and all their edges, either randomly or in a particular order. Then we are interested in comparing the flatness of the remaining network with a null model generated from it. For measuring the flatness, we need the expected number of transactions of all links, as well as their standard deviation. We use the following analytical approximation to estimate the null model distribution. Let $k_{i}^{out}$ denote the out-degree of node *i*. Similarly, $k_{j}^{in}$ is the in-degree of node *j*. Assuming that the probability of observing a link is independent of all other links, the probability of appearance of an edge from node *i* to *j* is independent of the connectivity of the rest of the edges, and it is given by

$$p_{ij}=\frac{k_{i}^{out}k_{j}^{in}}{N_{E}^{2}}$$ (5)

where *N*_*E*_ corresponds to the total number of edges in the network. Using the above probability, the expected number of transactions from *i* to *j* is

$$E_{ij}=N_{E}p_{ij}=\frac{k_{i}^{out}k_{j}^{in}}{N_{E}}$$ (6)

with standard deviation (since the distribution is binomial)

$$\sigma_{ij}=\sqrt{N_{E}p_{ij}(1-p_{ij})}$$ (7)

### Edge removal

Starting from the original observed network, we now remove links, according to the selected removal order. Similar to the case of node removal, we compare the remaining network with a null model. Here however, due to the eliminated links, which now have forbidden flows, both analytical approximations and simulations are challenging. Therefore, to obtain the desired distribution for the null model, we use the algorithm *MaxEnt* [53–56] to find the probability distribution that maximizes the Shannon entropy of the system given the node-level constraints (in- and out-degree) and the imposed edge-level constraints (no flow across certain edges). The distribution corresponding to maximum Shannon entropy is the least informative distribution, which in our case corresponds to the distribution of the null model [53, 54]. The Shannon Entropy is given by

$$H=-\sum_{ij}p_{ij}log(p_{ij})$$ (8)

and is a non-linear, convex function. We use non-linear programming to find:

$$\underset{p_{ij}}{\text{max}}\;H(p_{ij})$$

subject to:

$$\sum_{j}p_{ij}=k_{i}^{out}/N_{E}$$

$$\sum_{i}p_{ij}=k_{j}^{in}/N_{E}$$

$$0\leq p_{ij}\leq 1$$

$$p_{ij}=0,\;for\;any\;links\;ij\;in\;the\;set\;of\;constrained\;links$$

The expected number of transactions *E*_*ij*_ is then given by *E*_*ij*_ = *p*_*ij*_ * *N*_*E*_. Since MaxEnt cannot provide us with the standard deviation *σ*_*ij*_, we approximate it using Eq (7) and assuming that appearance of each edge is independent of other edges (thus it follows a binomial distribution).

## Supporting information

S1 File File containing supplemental information. (PDF)
