# Supplementary material for: Peer-to-peer lending and bias in crowd decision-making
Source: PLoS One. 2018 Mar 28;13(3):e0193007. doi: 10.1371/journal.pone.0193007 (PMC5873935; doi:10.1371/journal.pone.0193007)
Supplement: S1 File — (PDF) [file pone.0193007.s001.pdf]

Peer-to-Peer Lending and Bias in Crowd Decision-Making

Pramesh Singh,[1] Jayaram Uparna,[2] Panagiotis Karampourniotis,[3,4] Emoke-Agnes Horvat,[1,5] Boleslaw Szymanski,[4,6] Gyorgy Korniss,[3,4] Jonathan Z. Bakdash,[7,8,9] and Brian Uzzi[1*]

[1] Northwestern Institute on Complex Systems, Northwestern University, Evanston, IL, USA,

[2] Indian Institute of Management, Bangalore, 560076, Karnataka, India,

[3]Dept. of Physics, Applied Physics and Astronomy, Rensselaer Polytechnic Institute, Troy, NY, USA,

[4]Social and Cognitive Networks Academic Research Center, Rensselaer Polytechnic Institute, Troy, NY, USA,

[5]Department of Communication Studies, School of Communication, Northwestern University, Evanston, IL, USA,

[6]Dept. of Computer Science, Rensselaer Polytechnic Institute, Troy, NY, USA,

[7] US Army Research Laboratory, Aberdeen Proving Ground, MD, USA,

[8]US Army Research Laboratory South Field Element at the University of Texas Dallas, Dallas, TX, USA,

[9]Texas A&M University-Commerce, Commerce, TX, USA

[*]corresponding author

Email: uzzi@kellogg.northwestern.edu

Kiva Network

A visualization of the Kiva sub-networks from the year 2007 is shown in Figure 1.

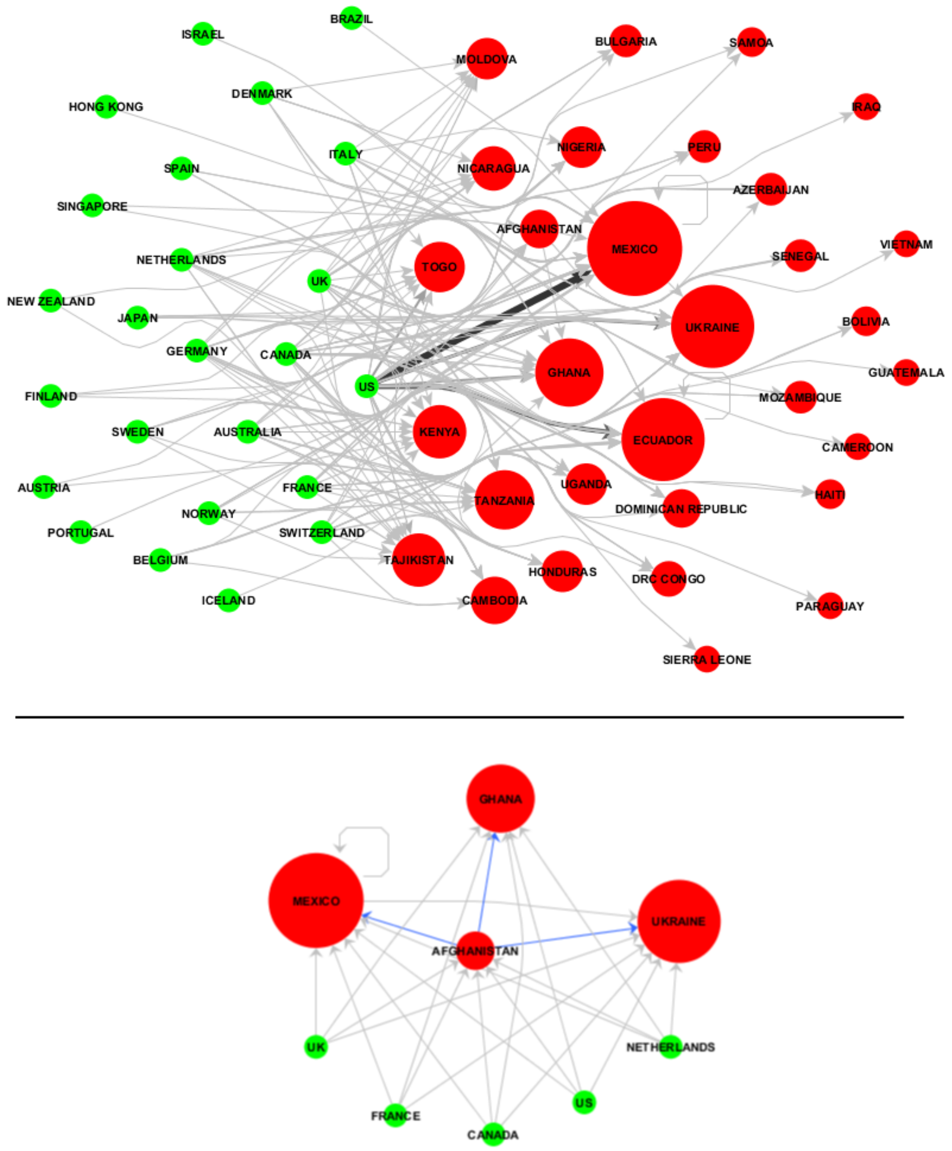

Figure 1. **Examples of Kiva sub-networks.** Top 200 links by number of transactions in the 2007 Kiva network (top). The borrower (lender) countries are colored red (green). The size of borrower country nodes is proportional to the received transactions; whereas, the lender countries are shown to be of the same size. Edge thickness is related to the number of transactions from lender country to borrower country. The figure contains only a subset of country–pairs for clarity. The ego-network of Afghanistan is for the same year (bottom). The outgoing links from Afghanistan have been colored differently following the same convention for node size and link thickness.

Example of Kiva Borrower and Lender Narratives

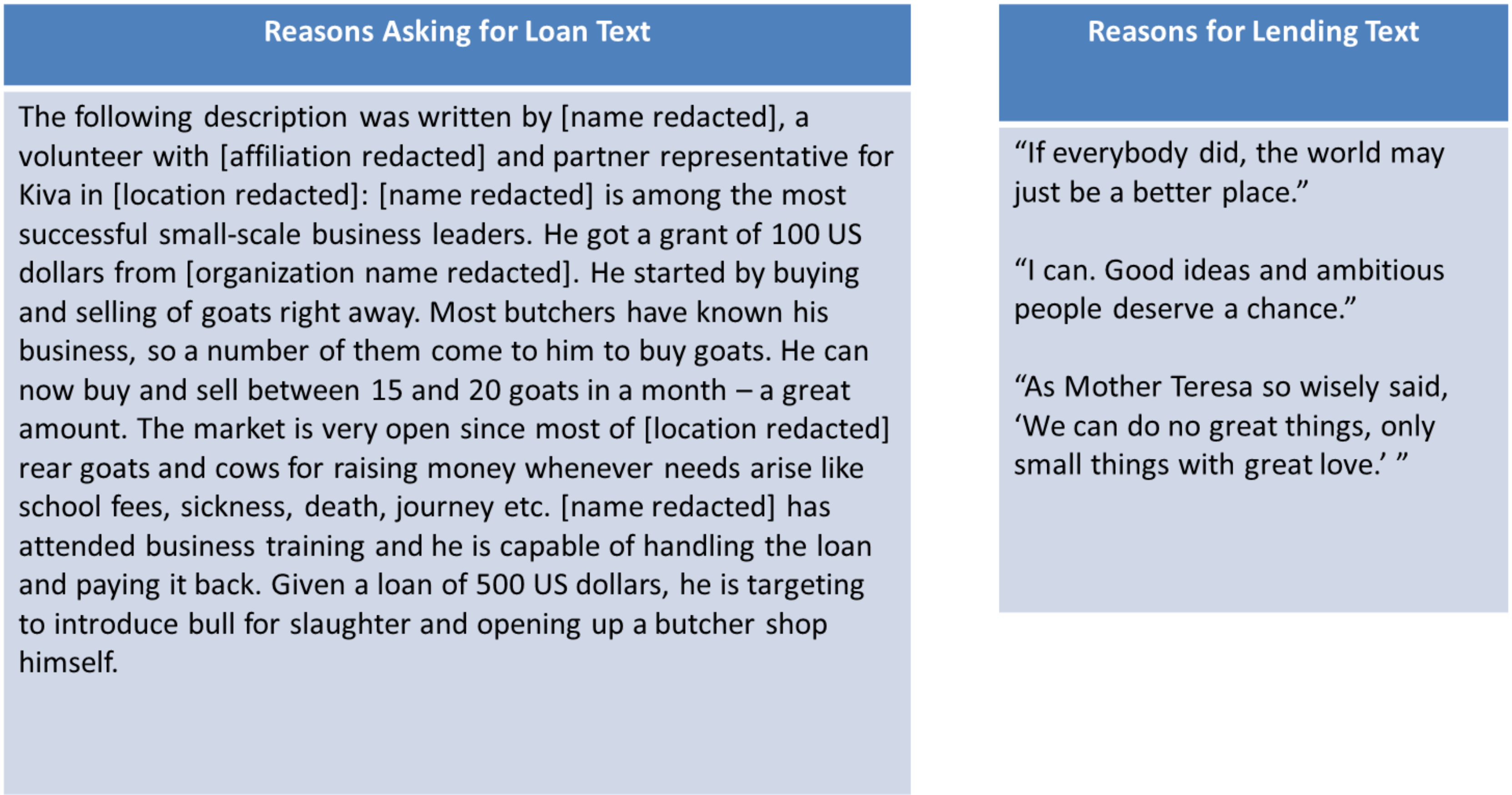

Figure 2. **A sample of representative borrowers and lenders' reasons for asking taken from Kiva's webpage.** Identifying information has been suppressed.

Flat World Null Model Robustness Checks

Time-window. In the null model of inter-country loan transactions, we randomize the transactions that took place in the same year. Similarly, we can also aggregate these transactions to any specified time interval and randomly rewire them within that window. For smaller time intervals, participating countries are not well represented and the null model's restrictive nature overly reduces differences between the observed and expected network. Nevertheless, in all cases we check the yearly, bi-annually, quarterly, and monthly time scales, and we observe statistically significant support for our claim that the world is getting less rather than more flat on the Kiva network (flatness is reduced by 10% in yearly and by 7% in bi-annual time scales).

Comparison of yearly z-score distributions. Figure 3 (i) shows the distribution of z-scores for each year in our sample. Figure 3 (ii) indicates that a statistically significantly larger fraction of country–pairs lie outside of the $\pm 2\sigma$ interval as the Kiva peer-to-peer network grows and ages (with the exception of the 2006–2007 period) (two sample year-to-year Kolmogorov-Smirnov [KS] tests). Further, the KS test statistic D_{ks} is larger away from the diagonal, which further demonstrates that the longer the time separation between years, the more dissimilarity in the z-score distribution. The range of z is widening on both positive and negative sides, which is indicative of abundance of high |z| with time. This broadening can be seen in terms of the distribution of |z|, which is shown in Figure 3 (iii).

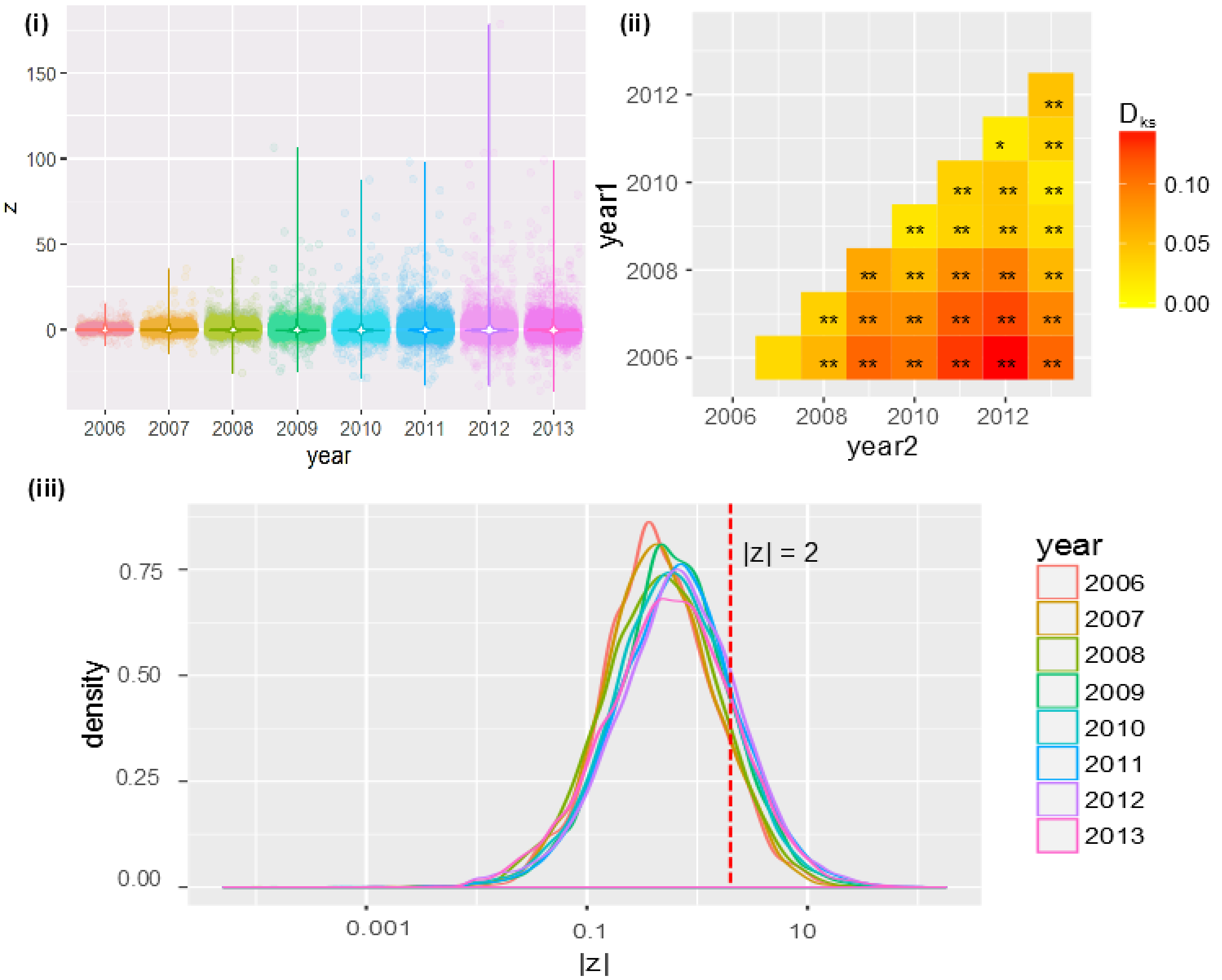

Figure 3. **Broadening of z-score distributions.** (i) Distribution of z-scores for each year shown by violin plots overlaid on the cloud of data points. It can be seen that the range of z-scores is becoming wider with time indicating a growing abundance of biased country–pairs. (ii) KS test statistic D_{ks} of the z-score distributions for every pair of years. Significance levels are indicated by stars ($^{*}p < 0.1$, $^{**}p < 0.05$) in each cell. All pairs of years show a significant ($p<0.1$ or $p<0.05$) difference except one (2006–2007), which is not significant. The color of each cell corresponds to the value of the KS statistic D_{ks}, which measures how far away the two distributions are. (iii) Probability distribution function of $|z|$ for each year with the dashed line showing the cut-off $|z| = 2$. Each curve corresponds to a particular year. This density plot shows that with time the distribution is shifting right, which indicates that a larger fraction of links is becoming biased (fraction beyond the $|z| = 2$ cut-off shown by the dashed line).

Categorical Dependent Variable

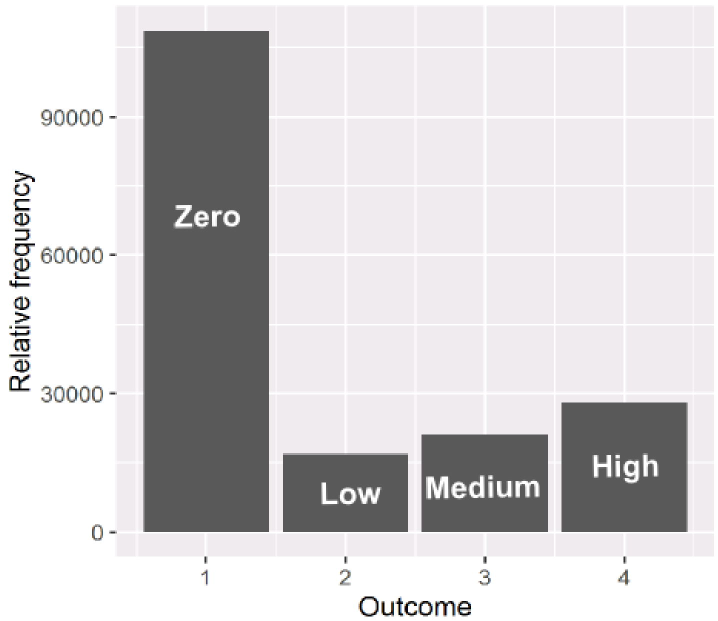

Figure 4. **Outcome variable for Kiva loans.** Quantiles of Y_{ijfy}. Outcomes (Q) represents zero (0 transactions), low (1 transaction), medium (2–7 transactions), and high volume (8–54,136 transactions) of transactions, respectively.

Table 1. **Descriptive statistics.**

Variable	Obs.	Mean	SD	Min	Max
Q (outcome)	174,468	1.82	1.16	1	4
GDP (pc) difference *(thousands USD)*	157,609	11.24	21.62	–47.96	122.16
Distance *(thousands of kms)*	164,803	8.55	4.55	0.010	19.95
Migration *(Millions)*	155,558	0.01	0.18	0	11.63
Colony	164,803	0.01	0.09	0	1

Table 2. Correlation matrix.

	Q (outcome)	GDP (pc) difference	Distance	Migration	Colony
Q (outcome)	1	. . .	. . .	. . .	. . .
GDP (pc) difference	0.42	1	. . .	. . .	. . .
Distance	–0.05	–0.03	1	. . .	. . .
Migration	0.06	0.03	–0.04	1	. . .
Colony	0.11	0.02	–0.03	0.09	1

Gravity Model

The results shown from the gravity model are qualitatively consistent with the ologit model in the main text. They show a positive and significant association of transaction with economic disparity, migration and colony, and a negative and significant association with geographical distance.

Here we model the number of transactions Y_{ijfy} from country i (lender) to country j (borrower) through the field partner f and in a given year y, using the gravity equation in the following way:

$$\log(Y_{ijfy}) = \log(G) + \alpha \log\left(\frac{GDP_i}{GDP_j}\right) + \beta \log(distance_{ij}) + \gamma \log(migration_{ji}) + \delta\ colony_{ij} + \varepsilon_{ijfy} \quad \textbf{(S1)}$$

where G is a constant, GDP_i and GDP_j are the per capita GDP of the lender and the borrower countries, *distance* is the geographical distance between i and j, *migration* is the migrant population of borrower country in the lender country, *colony* represents a colonial link between i and j (i being colonizer of j) and ε_{ijfy} is the error term. The model coefficient to be estimated is α, β, γ, and δ. We also include the fixed effects of lender country, borrower country, field partner, and year. Equation (S1) is the log transformed gravity equation (with fixed effects) where we included terms that capture the economic disparity between lender and borrower country (as the ratio of their per capita GDPs), distance, migration, and colonial past. The associated coefficients are estimated by performing a linear regression (see Table 5).

Table 3. **Descriptive statistics for gravity model.**

	Obs.	Mean	SD	Min	Max
log (transactions)	65,869	2.09	2.04	0	10.90
log (GDP ratio)	157,609	0.78	1.52	–4.44	5.36
log (distance)	164,803	1.93	0.77	–4.56	2.99
log (migration)	47,081	–7.61	3.10	–13.81	2.45
Colony	164,803	0.01	0.09	0	1

Table 4. **Correlation matrix for gravity model.**

	log (transactions)	log (GDP ratio)	log (distance)	log (migration)	colony
log (transactions)	1	. . .	. . .	. . .	. . .
log (GDP ratio)	0.3575	1	. . .	. . .	. . .
log (distance)	0.20	0.23	1	. . .	. . .
log (migration)	0.35	–0.10	–0.28	1	. . .
Colony	0.08	0.01	0.08	0.27	1

Table 5. **Gravity model regression.** Gravity model regression with number of transactions as the dependent variable.

N = 30216
$R^2 = 0.85$

log (transactions)	Coefficient	Robust SE	t	P>|t|
log (GDP ratio)	1.63	0.019957	81.53	0
log (distance)	–0.11	0.013652	–7.89	0
log (migration)	0.02	0.004096	4.84	0.001
Colony	0.12	0.017699	7.09	0
**Fixed effects**	. . .	. . .	. . .	. . .
Lender country	Yes	. . .	. . .	. . .
Borrower country	Yes	. . .	. . .	. . .
Field partner	Yes	. . .	. . .	. . .
Year	Yes	. . .	. . .	. . .

The results in Table 5 indicate that 1 unit increase in GDP ratio is associated with a 408% increase (fractional change = e^{α}-1) in number of transactions, 1 unit increase in distance is associated with a 10% decrease in transactions, 1 unit increase in migration is associated a 2% increase in transactions, and a presence of a colonial tie is associated with a 13% increase in transactions. Although the numerical estimates shown above cannot be compared exactly with the results obtained by the ologit model, their relationship to the bilateral transaction levels is similar.

We also look at the interaction between per capita GDP difference and migration by considering 10 quantiles of per capita GDP difference and migration (high = above median, low = below median) and modeling the number of transactions by the fixed-effect gravity model discussed above. The trend shown in Figure 5 is found to be qualitatively consistent with the ologit regression discussed in the main text.

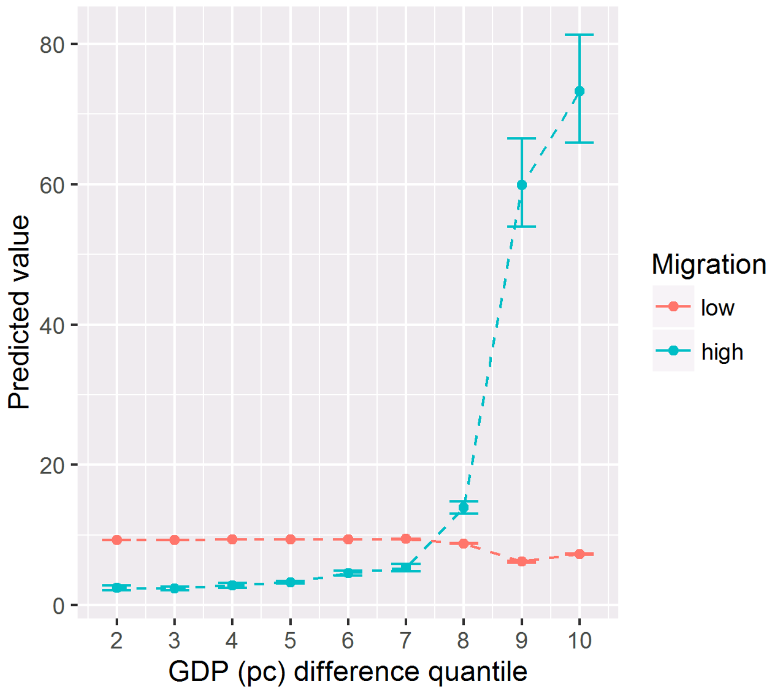

Figure 5. **Predicted transactions.** Predicted number of bilateral transactions as a function of per capita GDP difference quantile and level of migration. Error bars indicate ± 2 standard error (i.e., 95% confidence interval).

Global Financial Lending Flows: Kiva vs. Government Aid

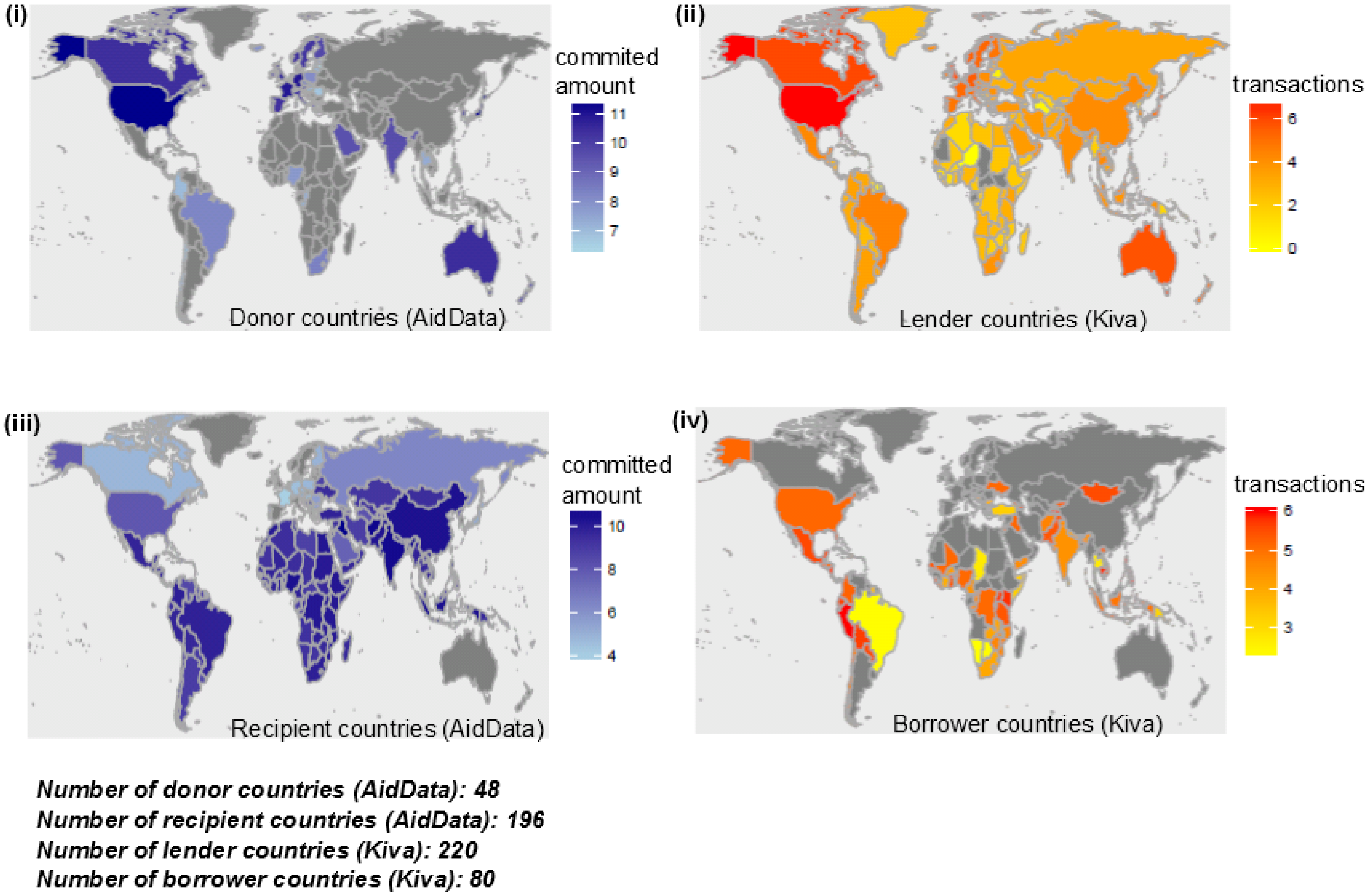

Figure 6. **Geographical coverage of Kiva and government aid.** (i) Donor countries by their total commitment amounts (USD), (ii) lender countries in Kiva by the total number of contributions made, (iii) recipient countries by total commitment amount (USD), and (iv) borrower countries in Kiva by the total number of contributions received. All values are aggregated sum from 2005–2013. The scale shown is logarithmic with a base of 10. The coverage patterns show a difference in the potential channels for capital flow. There are more participating lender countries on Kiva compared to number of donor countries from AidData in the same time period.

We compare the participation level of countries on Kiva and aggregated aid using data from AidData, (available at: http://aiddata.org/) from one country to another (only looking at country-to-country aid) for the same time period as Kiva (2005–2013). Figure 6 (i) and (iii) show the sum of commitment aid money (USD) given and received, respectively, by each donor country; and Figure 6 (ii) and (iv) show the total loan contributions made by the lenders in a lending country and total contributions made to the loans and borrower country, respectively.

The distribution of receivers of money through bilateral aid and through Kiva (by individual lenders) looks quite different. The Aid is distributed among recipient countries more uniformly whereas Kiva focuses mostly on fewer developing regions. The other distinguishing feature of Kiva is the global presence of individual lenders. The donor countries providing aid in the AidData are fewer in numbers (48) in comparison to Kiva lenders contributing from almost every country in the world (i.e., capital flow from "few-to-many" vs. "many-to-few"). Thus the Kiva dataset accounts for a much larger number of inter-country links that reach developing regions from developed regions.

Analysis of Government Aid Data

We construct a null model for the co-country aid network using data on international development aid [AidData] and extracting the yearly flow of country-to-country government aid (for the years 2005–2012). The null model is constructed by randomly rewiring the multi-edges in the network, where each (directional) edge represents an aid commitment made between a pair of countries. We preserve the total number of incoming edges and outgoing edges for each node (country). As in the case of the Kiva network (described in the text), by comparing the observed network with the null model we identify the biased links and compute the yearly flatness (as fraction of unbiased links in the given year). The flatness of the aid network is shown in Figure 7. We observe that the level of flatness in this network is lower than Kiva and does not follow a systematic trend. It can be inferred from Figure 7 that lending in the form of developmental aid by governments on an average is more biased than Kiva.

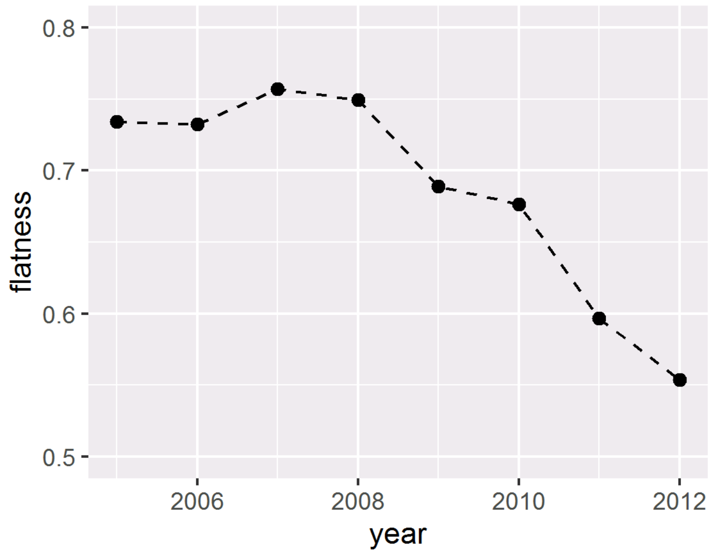

Figure 7. **Flatness of government aid network.** The level of flatness is low (compared to Kiva, which is between 90% and 80%) and increases between 2006 and 2007 and shows a decrease afterwards.

Next, to identify the potential factors associated with the observed bias, we model the level of aid using the fixed-effect ordered logistic regression given as follows:

$$Q_{ijy}^{(aid)} = \beta_1 GDP\ difference_{ijy} + \beta_2 Distance_{ij} + \beta_3 Migration_{ji} + \beta_4 Colony_{ij} + \varepsilon_{ijy}$$

(S2)

The fixed-effects of donor country, borrower country, and year were included in the model. The categorical outcome variable is constructed by using four quantiles (zero, low, medium, and high) of aid amount (shown in Fig. 8). The description of variables and their correlations are presented in Tables 6 and 7.

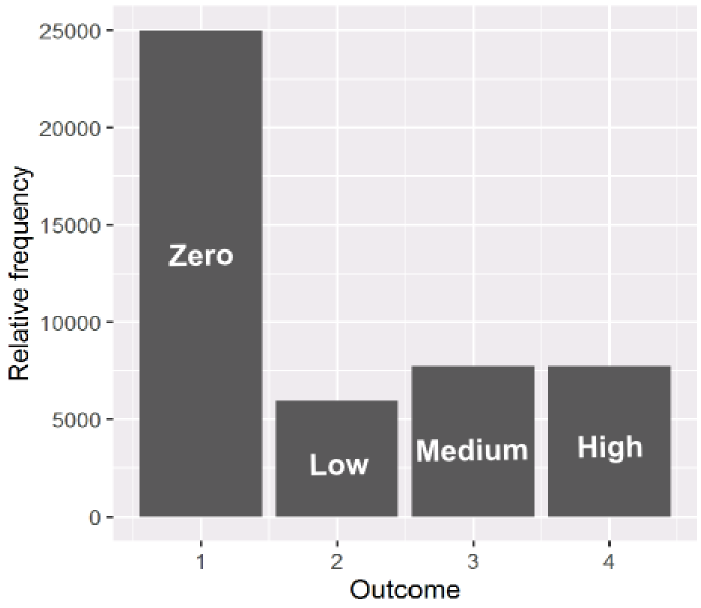

Figure 8. **Outcome variable for the government aid.** Relative frequency of levels of commitment amount (Zero: 0 USD; low: 8 USD–0.3 Million USD; medium: 0.3 Million USD–6.5 Million USD; high: 6.5 Million USD–11.1 Billion USD).

Table 6. **Descriptive statistics for government aid data.**

Variable	Obs.	Mean	SD	Min	Max
Outcome	46,456	2.89	2.105959	1	4
GDP (pc) difference *(thousands USD)*	39,938	27.66	19.2136	–44.72371	115.852
Distance *(thousands of kms)*	44,094	8.07	4.181782	0.0361766	19.95116
Migration *(Millions)*	42,813	0.02	0.1857959	0	11.63599
Colony	44,094	0.03	0.1620495	0	1

Table 7. **Correlation matrix for government aid data.**

	Outcome	GDP (pc) difference	Distance	Migration	Colony
Outcome	1	. . .	. . .	. . .	. . .
GDP (pc) difference	0.26	1	. . .	. . .	. . .
Distance	–0.14	–0.03	1	. . .	. . .
Migration	0.08	0.02	–0.05	1	. . .
Colony	0.16	–0.01	–0.02	0.06	1

The results of our ologit regression are reported in Table 8 (*N= 39,031*; *pseudo* R^2*= 0.4068* for the final model) and show that similar to lending in Kiva, government aid is also driven by the same exogenous variables with the exception of GDP difference, which in the case of government aid was not found to be significant. The effect of migration and colonial past, as reflected by very high odds ratios, are much stronger in this case.

Table 8. **Fixed-effect ologit estimates of levels of aid between countries.** Odds ratio reported for 4 levels of commitment amount as defined.

	Odds ratio Model 1	Odds ratio Model 2	Odds ratio Model 3	Odds ratio Model 4
GDP (pc) difference	0.99	0.99	0.99	0.99
Distance	. . .	0.77^{**}	0.77^{**}	0.77^{**}
Migration	. . .	. . .	5.21^{**}	2.52^{**}
Colony	. . .	. . .	. . .	12.65^{**}
AIC:	62,907.5	58,361.99	58,284.72	57,371.25
BIC:	62,976.26	58,430.56	58,353.3	57,439.83
**Fixed effects:**	. . .	. . .	. . .	. . .
Year	Yes	Yes	Yes	Yes
Donor country	Yes	Yes	Yes	Yes
Recipient country	Yes	Yes	Yes	Yes

$^{**}p < 0.05$

Node and Link Removal Simulations

Because of the large number of nodes and links in the network, simulations for all of them were computationally infeasible; therefore, we applied a well-accepted numerical method to approximate the simulation results. Here, we show that the network estimated by simulation or analytical methods show close agreement with one another for 2006. A comparison between simulations and the numerical approximation (Figures 9 and 10) shows that the agreement between the approximation and degree preserving simulation is good. Since this approach overestimates the standard deviation slightly because of the small contribution from node degree not being preserved exactly but only on an average, we see that the flatness obtained by analytical approximation is larger in a systematic way even though the magnitude of the difference is small. Moreover, since the analytical method always overestimates the flatness (and this is true for all node/link removal methods), it only shifts the flatness measure by a small amount and does not affect the overall trend of flatness change with respect to removal (Figures 9 and 10).

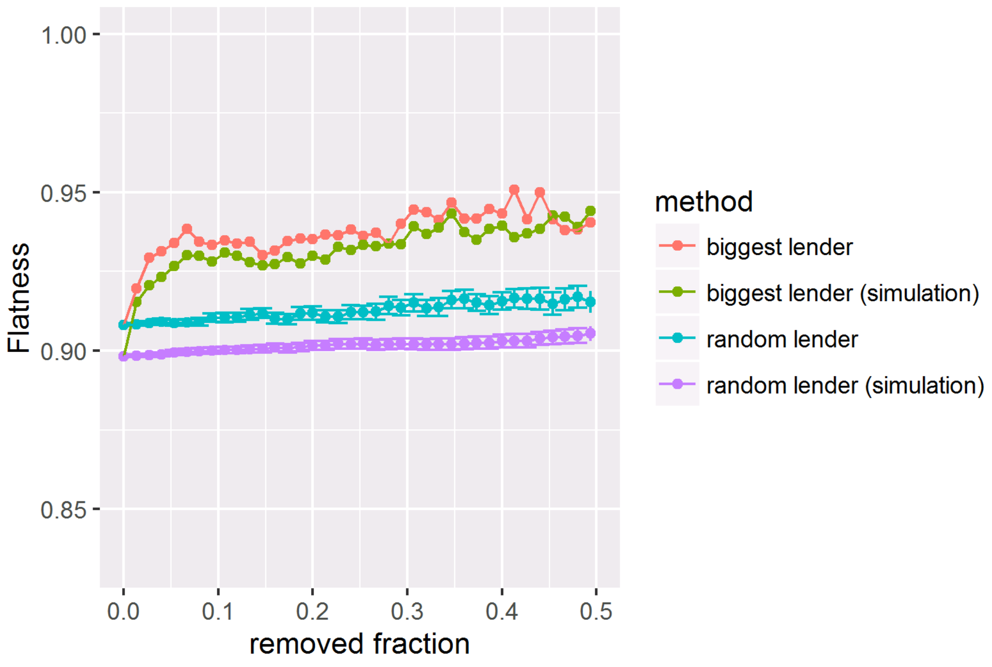

Figure 9. **Comparison between simulation and analytical approximation for node removal for 2006 (for random and degree based removals).** Results show good agreement between simulation and analytical approximation. The analytical approximation by construction overestimates the flatness as explained in the text. The error bars correspond to ± 2 standard error for the random removal case.

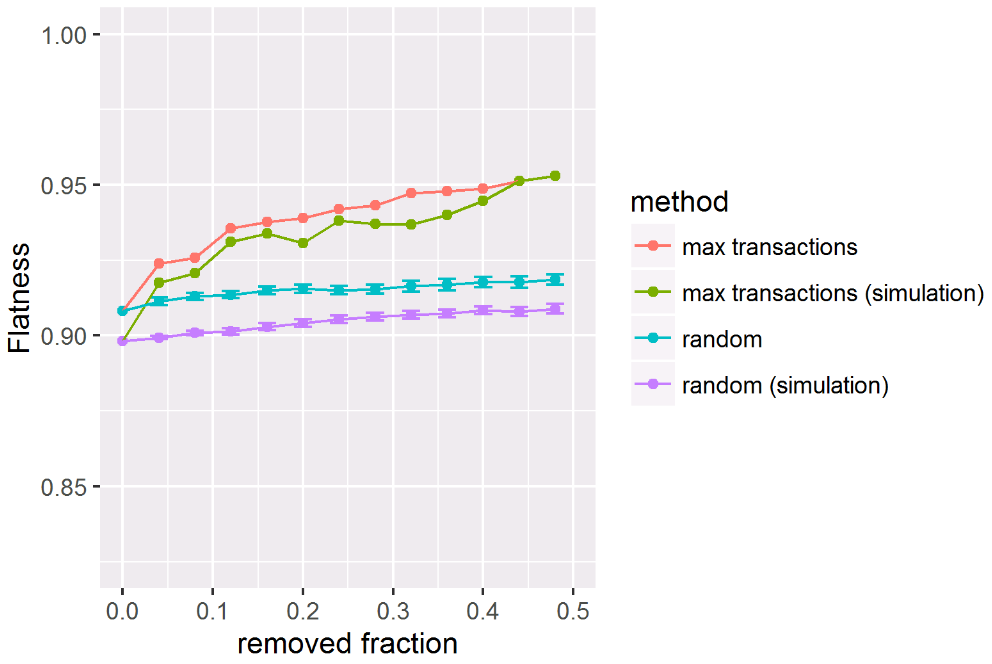

Figure 10. **Comparison between simulation and maximum entropy method for link removal for 2006 (for random and transaction-based removals).** Results show a good agreement in the trend between simulation and maximum entropy method. The maximum entropy method overestimates the flatness as explained in the text. The error bars correspond to ± 2 standard error for the random removal case.

Targeted Link Removal

Figure 11 shows that there are slightly more positively biased links than negatively biased for all years. This has an effect when links are removed by maximum and minimum z-scores. Since removal of biased links have a stronger effect on flatness, the curve corresponding to minimum z-score becomes flatter before the one corresponding to maximum z-score (see Figure 7, main text). The discrepancy is larger for the year 2006 where there are much fewer negatively biased links than positively biased links. The flatness continues to increase for link removal based on maximum transactions beyond maximum z-score and minimum z-score based removal. This can be attributed to the fact that the number of transactions are highly correlated with the absolute z-score as shown in Figure 12. Removal according to maximum or minimum z-score starts by targeting either positively or negatively biased links, respectively; whereas, removal by transaction has the advantage of potentially targeting positively as well as negatively biased links.

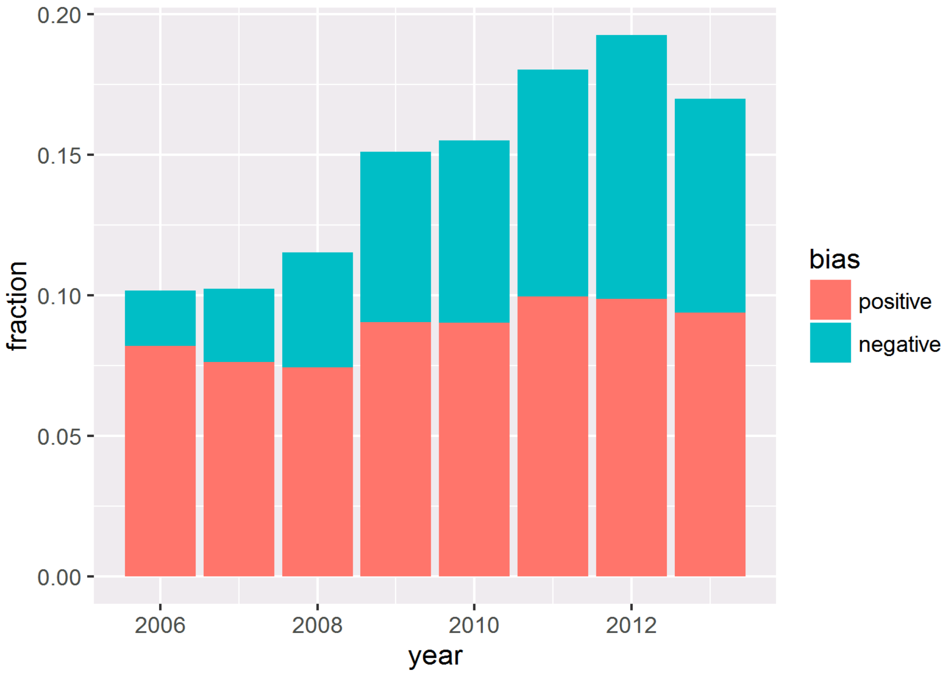

Figure 11. **Positively vs. negatively biased fraction of links.** Fraction of positively ($z > 2$) biased (red), and negatively ($z < -2$) biased links (blue) for the years 2006–2013. The figure shows a slightly larger proportion of positively than negatively biased links.

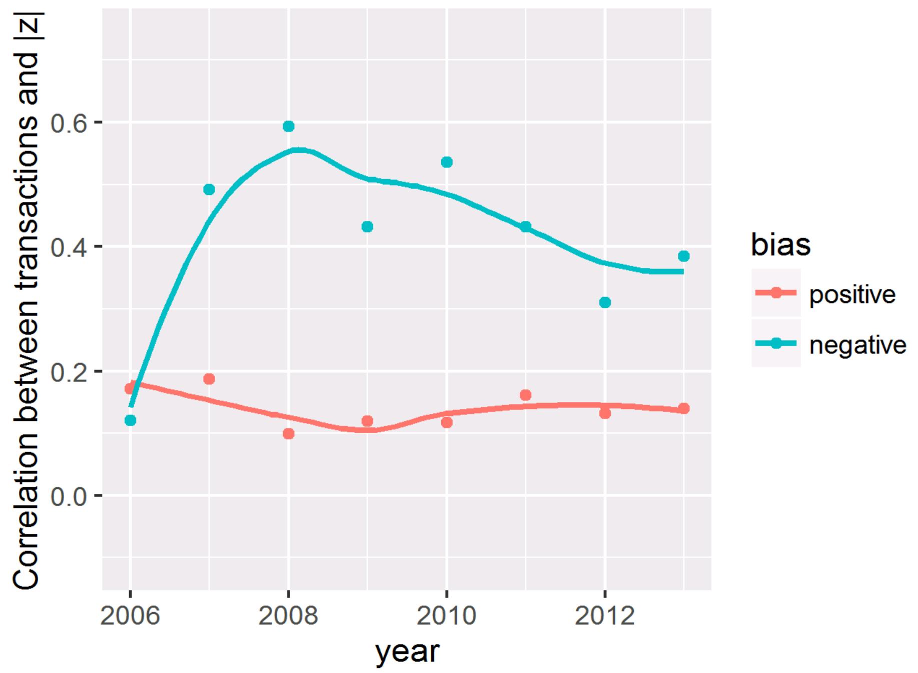

Figure 12. **Correlation between the number of transactions and absolute value of z-scores.** Linear correlation between absolute value of z-score for the biased pairs of countries in the network (computed separately for positively ($z > 2$) and negatively ($z < -2$) biased links) and the number of transactions between a pair of countries for years 2006–2013. Red and blue points correspond to positive and negative z-scores. The number of transactions seem to be correlated with both positively and negatively biased links. Thus, the removal of links with maximum transactions has a similar effect on the system flatness as removal of highly biased (positive or negative) links.
